# Transcriptome and Biochemical Analyses Revealed a Detailed Proanthocyanidin Biosynthesis Pathway in Brown Cotton Fiber

**DOI:** 10.1371/journal.pone.0086344

**Published:** 2014-01-21

**Authors:** Yue-Hua Xiao, Qian Yan, Hui Ding, Ming Luo, Lei Hou, Mi Zhang, Dan Yao, Hou-Sheng Liu, Xin Li, Jia Zhao, Yan Pei

**Affiliations:** Biotechnology Research Center, Southwest University, Beibei, Chongqing, China; University of New South Wales, Australia

## Abstract

Brown cotton fiber is the major raw material for colored cotton industry. Previous studies have showed that the brown pigments in cotton fiber belong to proanthocyanidins (PAs). To clarify the details of PA biosynthesis pathway in brown cotton fiber, gene expression profiles in developing brown and white fibers were compared via digital gene expression profiling and qRT-PCR. Compared to white cotton fiber, all steps from phenylalanine to PA monomers (flavan-3-ols) were significantly up-regulated in brown fiber. Liquid chromatography mass spectrometry analyses showed that most of free flavan-3-ols in brown fiber were in 2, 3-trans form (gallocatechin and catechin), and the main units of polymeric PAs were trihydroxylated on B ring. Consistent with monomeric composition, the transcript levels of flavonoid 3′, 5′-hydroxylase and leucoanthocyanidin reductase in cotton fiber were much higher than their competing enzymes acting on the same substrates (dihydroflavonol 4-reductase and anthocyanidin synthase, respectively). Taken together, our data revealed a detailed PA biosynthesis pathway wholly activated in brown cotton fiber, and demonstrated that flavonoid 3′, 5′-hydroxylase and leucoanthocyanidin reductase represented the primary flow of PA biosynthesis in cotton fiber.

## Introduction

Naturally colored cotton is an important raw material for ecological textiles. With naturally colored cotton, textile manufacturers can eliminate dyeing during processing, and significantly reduce processing costs, environmental pollutions and chemical residues in fabrics [1], [2]. In addition, naturally colored cotton may have lower flammability and higher ultraviolet protection value compared to traditional white cotton [3], [4]. Brown cotton fibers with different shades are most widely used in the modern colored cotton industry. A wealth of information has been obtained about the chemical properties and biosynthesis pathway of brown pigments in cotton fiber, suggesting that these pigments belonged to proanthocyanidins (PAs) [5]–[9]. However, exact chemical properties of PA pigments and details of the PA biosynthesis pathway in brown cotton fiber are still to be elucidated.

**Figure 1:**
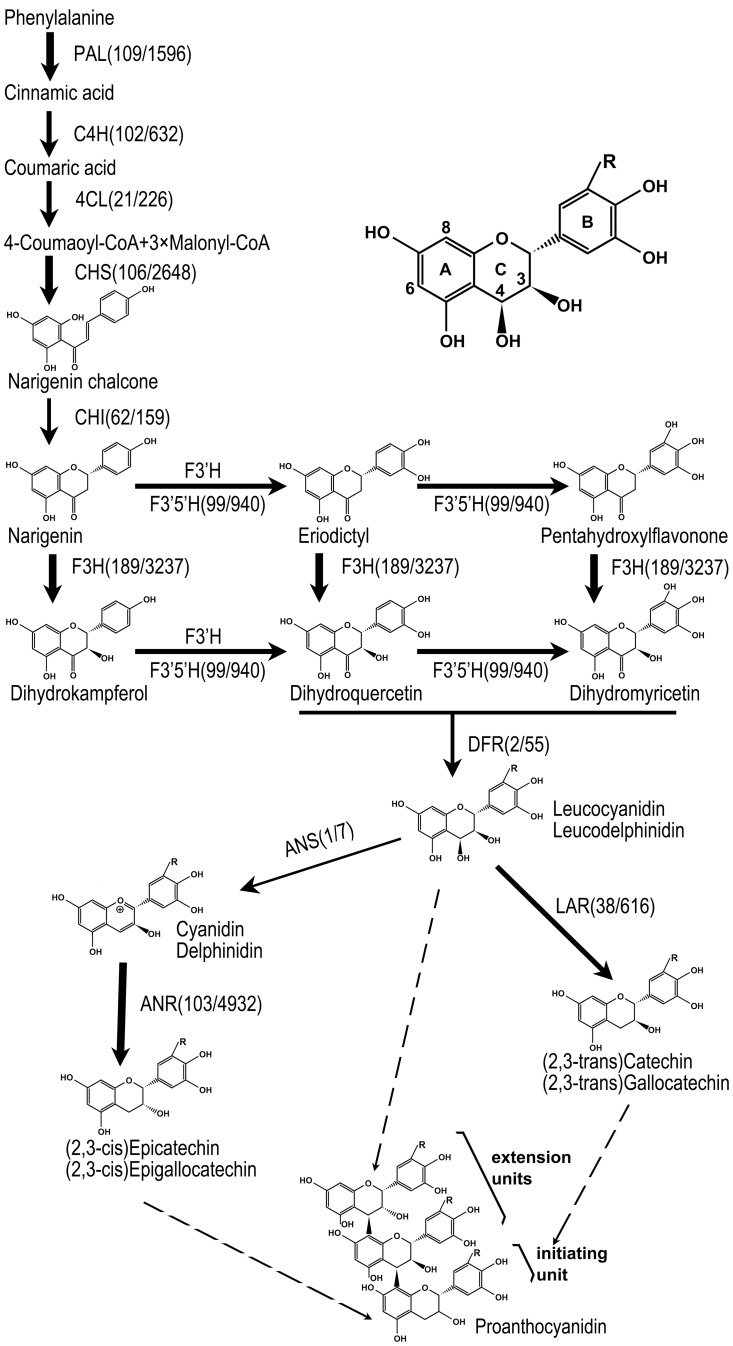
The detailed PA biosynthesis pathway in brown cotton fiber. A typical PA monomer is depicted in the up right (R = H or OH). Solid arrows represent reactions from substrates to products with corresponding synthases indicated. The arrow line thickness roughly reflects the expression levels of corresponding synthases and putative flow rates in these steps. Expression levels are classified into 4 categories according to transcript abundance detected in the DGE analysis, i.e. low, moderate, high and very high expression (2∼20, 20∼200, 200∼1000 and over 1000 TPM, respectively). Transcript levels (in TPM) of various PA synthases in white/brown fibers are indicated. Dashed arrows indicate the monomeric origins of oligomeric or polymeric PAs. PA synthases are abbreviated as in Table 1.

PAs, also known as condensed tannins, are widely distributed in plants with various functions such as pigments in seed coat and protectants against herbivores and microbes [10], [11]. PA is also an important factor affecting mouthfeel, contributing the bitter flavor and astringency to our daily foods and beverages [12]. In addition, PA’s antioxidant and anti-inflammatory properties make it a potential chemopreventive and chemotherapeutic agent for some human diseases, including cancers [12], [13]. Chemically, PAs are oligomers or polymers of polyhydroxy flavan-3-ol units. PA polymers are synthesized presumably by adding flavan-3, 4-diol (leucoanthocyanidin) molecules to an initiating flavan-3-ol unit or the terminal unit of a flavan-3-ol chain (Figure 1) [10], [11], [14]. Both flavan-3-ols and flavan-3, 4-diols are synthesized through plant flavonoid pathway. The details of this pathway vary with tissues and species and determine PA compositions in different plants [15].

In attempt to clarify the details of PA biosynthesis pathway in brown cotton fiber, we performed a digital gene expression (DGE) analysis to compare the gene expression profiles in brown and white fibers. A total of 24 PA synthase genes were identified to be significantly up-regulated in brown fiber. Furthermore, we determined the chemical properties of PAs in brown fiber by using liquid chromatography-mass spectrometry (LC-MS) method. It was found that the majority of PA initiating units consisted of gallocatechin and the main extension units in brown fiber were trihydroxylated on B ring. These results demonstrated a detailed PA pathway involved in the brown pigmentation in cotton fiber, which may be essential to manipulate the biosynthesis of pigment and other flavonoids in cotton fiber.

## Materials and Methods

### RNA Extraction and Solexa Sequencing

A recombination inbred line (RIL) population derived from the cross between white fiber cotton cultivar Yumian No.1 and brown fiber line T586 was constructed as described [16]. Total RNAs were extracted from fibers of 14 days post anthesis (DPA) using a modified CTAB method [17]. Equal amounts of total RNAs from each 10 white and brown fiber RILs were mixed to form two RNA bulks (WCF and BCF, respectively). DGE analyses of RNA bulks were performed in BGI-Shenzhen using standard procedure (Shenzhen, Guangdong, China) [18], [19]. Briefly, biotin-labeled oligo d(T) primer was employed to initiate the synthesis of double-stranded cDNA. After *Nla*III restriction, the 3′ cDNA ends were separated by magnetic method and linked to an adaptor containing a *Mme*I recognition site. After *Mme*I digestion and ligation to the second adaptor, the tags were sequenced on an Illumina Genome Analyzer (Illumina, Inc., San Diego, CA) using Solexa technology. The raw sequence data were deposited in NIH Sequence Read Archive under the accession number SRP033354. Sequenced tags were annotated by aligning with *Gossypium hirsutum* unigenes and singleton ESTs (ftp://ftp.ncbi.nih.gov/repository/UniGene/Gossypium-hirsutum/Ghi.seq.uniq.gz, 2010) [20]. Differentially expressed genes (DEGs) in WCF and BCF bulks were identified according to the frequency of corresponding tags by setting a cutoff on a false discovery rate (FDR) <0.001 and a frequency ratio of BCF to WCF >2 or <0.5. Interested unigenes and ESTs were mapped to cotton D genome [21] by blast searching in phytozome (http://www.phytozome.net/search.php) [22].

### Quantitative RT-PCR

Quantitative RT-PCR (qRT-PCR) was employed to detect the expression levels of predominant PA synthase genes in each 10 brown and white fiber RILs. The investigated genes and corresponding primers were listed in table 1. The histone 3 and elongation initiation factor 5 genes from cotton were amplified as RNA standard [23], [24]. PCRs were performed on a CFX96™ real-time PCR detection system with SYB Green supermix (Bio-Rad, CA, USA). The thermocycling parameters were as follows: 95°C, 2 min, 40 cycles of 95°C, 10 s and 57°C, 20 s. A standard melting curve was added to monitor the specificity of PCR products. The reactions were duplicated for 3 times and data were analyzed using the software Bio-Rad CFX Manager 2.0 provided by manufacturer.

**Table 1:** The real-time PCR primers used in this study.

Genes in D genome	PA synthases^1^	Primers	Sequences (5′ to 3′)
Gorai.002G248000	PAL	PAL-F	AGCTTGGAACTGGGTTGTTG
		PAL-R	AGCACCATTCCAACCCTTTA
Gorai.013G271700	C4H	C4H-F	TTTGGGTCGTTTGGTACAGA
		C4H-R	AAAATTGCCTTGGCTTAGCA
Gorai.003G052100	4CL	4CL-F	AAGGTGCACTTTGTTCATGC
		4CL-R	CGTTGCAATTTAAAAGCCAAAT
Gorai.011G161200	CHS	CHS-F	CAGGAGAAGGACTGGAGTGG
		CHS-R	AGCAGCAACACTATGGAGCA
Gorai.013G023400	CHI	CHI-F	ATGGAGTTTCTCCTCCAGCA
		CHI-R	GGTTTTTCACTGTCGACTCCA
Gorai.008G062900	F3H	F3H-F	CTGAAGAAGCTGGCCAAAGA
		F3H-R	TGCAAGGATTTCCTCCAATG
Gorai.008G198200	F3′H	F3′H-F	GCTGATGTTAGGGGCAATGA
		F3′H-R	CTCACCATGAAACGACAACG
Gorai.001G134900	F3′5′H	F3′5′H-F	AAACATGGATGAGGCCTTTG
		F3′5′H-R	GCAAGGGATGTGCTTAGGAA
Gorai.009G200600	DFR	DFR-F	CATGTTCGTAGGAGCTGTCG
		DFR-R	GGTAGGCACTCAATTGTTGAAA
Gorai.008G186500	LAR	LAR-F	GAATGAGCCATTCCGAACAT
		LAR-R	GCTTCGACTACTGGCTTTGG
Gorai.004G205900	ANS	ANS-F	GCCACCGAAGGATAAGATCA
		ANS-R	TGGGTCTTCCTGAACAGCTT
Gorai.009G175500	ANR	ANR-F	TGGGATCGAGGAAATCTACG
		ANR-R	ACCATAATCATTGGGGAAGC
	Histone 3	His-F	GAAGCTGCAGAGGCATACC
		His-R	CTACCACTACCATCATGGC
	Elongation initiation	Eif5-F	GGTTGCCATTGTGCAAGGA
	factor 5	Eif5-R	CCGTAGGTGAGCGTTAATCAGA

1 4CL, 4-coumarate:CoA ligase; ANR, anthocyanidin reductase; ANS, anthocyanidin synthase; C4H, cinnamate 4-hydroxylase; CHI, chalcone isomerase; CHS, chalcone synthase; DFR, dihydroflavonol 4-reductase; F3H, flavone 3-hydroxylase; F3′H, flavonoid 3′-hydroxylase; F3′5′H, flavonoid 3′5′-hydroxylase; LAR, leucoanthocyanidin reductase; PAL, phenylalanine ammonia-lyase.

### Extraction and Purification of Cotton Fiber PAs

PAs were extracted from developing brown cotton fibers and purified as described [25]. Around 10 g fibers harvested from bolls of 20 DPA were ground to fine powder in liquid N_2_, and extracted in 50 ml 80% aqueous acetone twice at 4°C for 2 h. Solutions were centrifuged at 10 000 rpm for 5 min. Supernatants were combined and acetone was evaporated under vacuum at 35°C. Two-microliter aliquots of the remaining aqueous solution were applied to a packed Supelco Discovery DPA-6S polyamide cartridge (500 mg, Sigma-Aldrich Chemie Inc.). After washing with 5 ml 30% methanol, PAs were eluted with 2 ml 90% N, N-dimethylformamide. Finally, eluates were evaporated to dry under vacuum, re-suspended in methanol, and subjected to LC-MS analyses.

### LC-MS Analysis

To detect monomeric flavan-3-ols in the purified PAs, LC-MS analyses were performed on LC-MS 2010A system (Shimadzu, Japan) with an Xtimate C18 column (2.1×150 mm, 5 µm, Welch Materials, Inc., Shanghai, China). Solvent A was water: formic acid (99∶1, v/v), and solvent B was acetonitrile: formic acid (99∶1, v/v). Around 5 µg PAs were injected and eluted with a gradient of solvent B to A at a flow rate of 0.2 ml/min. After 3-min isocratic elution in 90% solvent A and 10% solvent B, solvent B concentrations increased from 10% to 30% (v/v) in 12 min, followed 15-min washing with 100% of B and 3-min re-equilibration in 10% of B. Positive ions were monitored in a selected ion monitoring mode. Monomeric flavan-3-ols were identified according to the typical ions and retention times of authentic standards. Catechin and epicathechin with [290+H]^+^ ions were eluted at 8.64 and 14.12 min, while gallocatechin and epigallocatechin with [306+H]^+^ ions were detected at 3.92 and 6.62 min, respectively. Flavan-3-ol amounts were calculated by reference to the standard curves of authentic standards.

To elucidate the chemical properties of PA extension unit, purified PAs were hydrolyzed by acid butanol method [26]. PAs were mixed with equal volume (50 µl) of concentrated HCl: butanol (1∶9, v/v) and incubated at 100°C for 1 h. The hydrolytes were evaporated to dry under vacuum, re-suspended in 100 µl aqueous methanol (15%, v/v), and subjected to LC-MS analysis. The column and solvents were the same as those used to detect monomeric flavan-3-ols, while the gradient profile was as follows: 0 to 3 min, 10% of solvent B; 3 to 20 min, 10% to 40% of B; 20 to 40 min 100% of B; and 20 to 23 min, 10% of B for re-equilibration. Typical ions and retention times for delphinidin, cyanidin and pelargonidin (Indofine, Hisllsborough, NJ, USA) were [304+H]^+^ at 14.81 min, [288+H]^+^ at 16.73 min, and [272+H]^+^ at 18.33 min, respectively. Anthocyanidins were quantified according to the areas of typical peaks by reference to the standard curves of corresponding standards.

## Results

### Digital Gene Expression (DGE) Analysis of Brown and White Cotton Fibers

**Figure 2:**
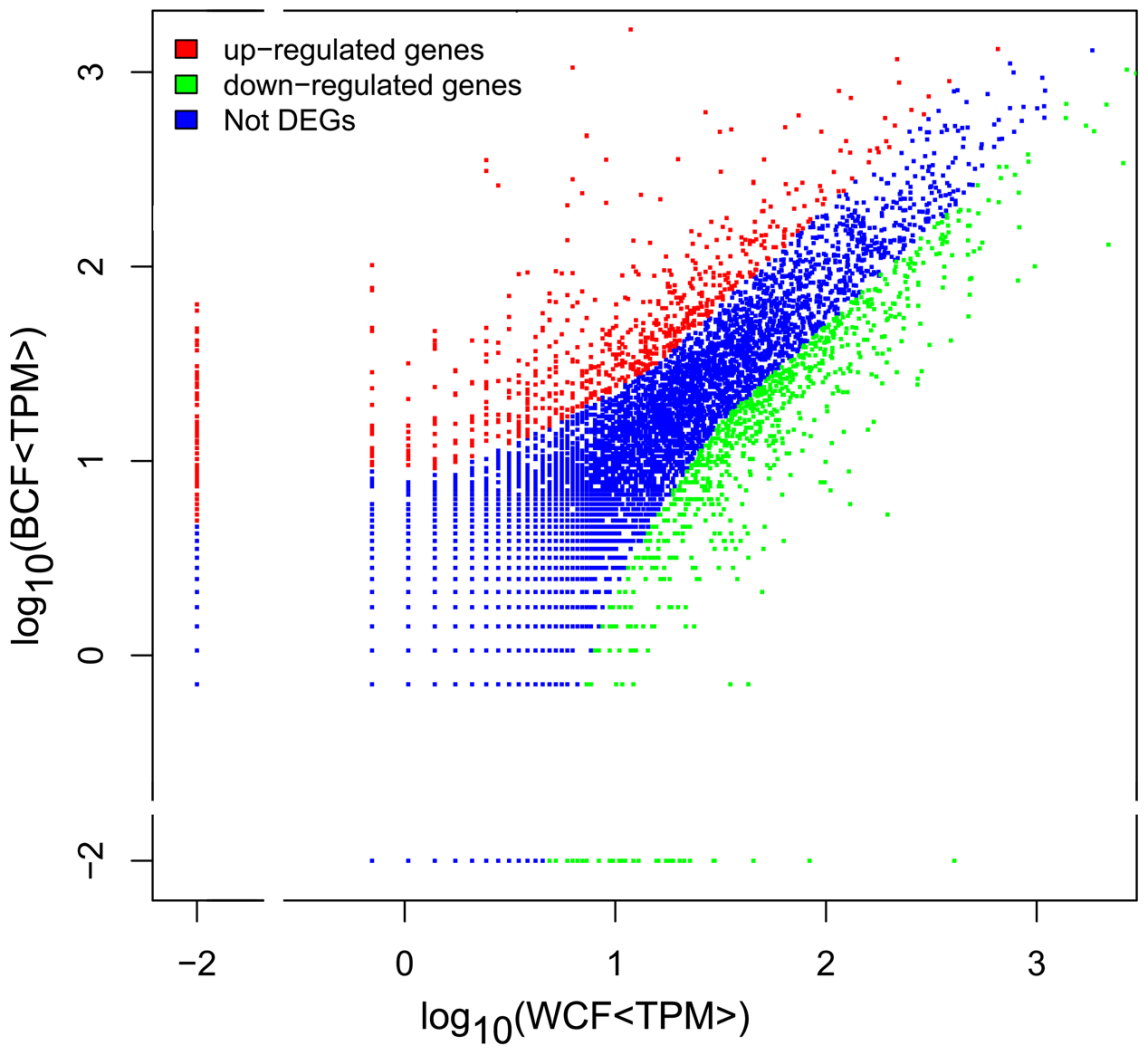
Distribution of gene expression levels in white and brown fibers. Gene expression level was standardized to transcripts per million (TPM). When no transcript was detected in a certain RNA bulk, the expression level was set to 0.01 TPM. WCF and BCF, white and brown cotton fiber RNA bulk, respectively. Differentially expressed genes (DEGs) include up-regulated and down-regulated genes with expression level ratio (BCF/WCF) >2 and <0.5, respectively, and the false discovery rate (FDR) <0.001.

Accumulation of flavonoids and pigments in fibers may retard the fiber development and reduce the final fiber quality and yield [9], [27], [28]. To dissect the molecular basis of pigment biosynthesis and its effect on fiber development, gene expression profiles in brown and white cotton fibers (BCF and WCF) were compared by DGE analysis. More than 3 million tags were generated from the developing fibers, including 2 860 036 and 2 913 186 clean tags in BCF and WCF libraries, respectively. Among these, 35664 (31.26%) distinct tags in BCF and 29048 (30.27%) in WCF were unambiguously mapped to a certain *G. hirsutum* unigene or singleton EST (ftp://ftp.ncbi.nih. gov/repository/UniGene/Gossypium-hirsutum/Ghi.seq.uniq.gz, 2010). Gene expression levels in BCF and WCF bulks were compared according to the corresponding tag frequencies. A total of 2079 differentially expressed genes (FDR<0.001) were identified, among which 1165 genes were up-regulated (expression level ratio BCF/WCF>2) and 914 were down-regulated (BCF/WCF<0.5) in brown fibers (Figure 2).

Gene ontology (GO) analysis suggested that the cellular components of vehicles (including membrane-bounded vesicle and cytoplasmic vesicle) and membrane were most significantly over-represented in differentially expressed genes (DEGs) between white and brown fibers (Table 2). The most significantly over-represented Kyoto Encyclopedia of Genes and Genomes (KEGG) pathways in DEGs were oxidative phosphorylation and ubiquitin mediated proteolysis (Table 3). In addition, some DEGs were related to the physiological processes involved in regulation of fiber development, such as reactive oxygen [29] and ethylene [30] (Table 4). These data indicated that the brown fiber gene (*Lc1*) and/or pigment accumulation in cotton fiber may affect multiple aspects in fiber development other than fiber coloration.

**Table 2:** Over-represented GO cellular components in DEGs of brown and white fibers.

Gene ontology term	Clustered gene and frequencyin DEGs (989)	Clustered gene and frequencyin reference genes (25492)	P-value	GO ID
membrane-bounded vesicle	226, 22.9%	4474, 17.6%	0.003	GO:0031988
cytoplasmic membrane- bounded vesicle	225, 22.8%	4461, 17.5%	0.004	GO:0016023
vesicle	226, 22.9%	4503, 17.7%	0.005	GO:0016020
membrane	328, 33.2%	6939, 27.2%	0.005	GO:0031982
cytoplasmic vesicle	225, 22.8%	4489, 17.6%	0.006	GO:0031410

**Table 3:** Over-represented KEGG pathways in DEGs of brown and white fibers.

Pathway	DEGs with pathway annotation (1337)	Reference genes with pathway annotation (45320)	P value	Pathway ID
Oxidative phosphorylation	57, 4.26%	1123, 2.48%	0.000	ko00190
Ubiquitin mediated proteolysis	49, 3.66%	985, 2.17%	0.000	ko04120

**Table 4:** ROS- and ethylene- related DEGs of brown and white fibers.

Genes	WCB(TPM)	BCF(TPM)	P-Value	FDR	Homologous proteins
ROS related					
Ghi.16954	0.01	8.39	4.73E-08	4.93E-07	cytosolic ascorbate peroxidase
Ghi.9579	1.03	16.78	7.56E-05	4.16e-04	Cu/Zn superoxide dismutase
Ghi.16311	4.12	25.87	5.37E-07	4.79E-06	Cu/Zn superoxide dismutase
Ghi.9542	89.94	42.31	1.24E-12	2.23E-11	Mn superoxide dismutase
Ghi.9864	106.76	241.95	4.26E-14	1.04E-12	Peroxidase precursor
Ghi.16267	5.15	0.01	3.53E-05	2.11e-04	Peroxidase precursor
Ghi.16323	55.95	13.99	1.58E-18	5.42E-17	peroxisomal targeting signal 2 receptor
Ethylene related					
Ghi.16253	32.95	68.18	1.91E-09	2.44E-08	1-aminocyclopropane-1- carboxylate synthase
Ghi.16693	6.52	0.01	2.29E-06	1.80E-05	1-aminocyclopropane-1- carboxylate oxidase
Ghi.4454	46.34	18.53	2.89E-09	3.59E-08	ethylene-insensitive3 protein
Ghi.16250	239.26	95.8	1.28E-41	7.92E-40	ethylene-responsive element binding protein ERF2
Ghi.22814	0.01	5.24	2.63E-05	1.64 E-04	ethylene-responsive element binding protein ERF2
Ghi.9501	5.49	0.01	1.78E-05	1.15E-049	Ethylene-responsive transcription factor
Ghi.2915	0.01	5.24	2.63E-05	1.64 E-04	Ethylene-responsive transcription factor
Ghi.13085	22.31	46.5	5.85E-07	5.16E-06	ethylene-responsive element-binding factor

### Expressions of PA Synthase Genes in White and Brown Fibers

In higher plants, PAs are synthesized through the flavonoid pathway from phenylalanine to flavan-3-ols (Figure 1) [10], [11], [14], [15]. By DGE analysis of brown and white cotton fibers, a total of 34 PA synthase genes were identified (Table 5). Among these genes, 24 were significantly up-regulated in brown fiber, but none was significantly down-regulated. These up-regulated genes encoded 3 phenylalanine ammonia-lyases (PAL), 2 cinnamate 4-hydroxylases (C4H), 1 4-coumarate:CoA ligase (4CL), 6 chalcone synthases (CHS), 1 chalcone isomerase (CHI), 2 flavanone 3-hydroxylases (F3H), 2 flavonoid 3′, 5′-hydroxylases (F3′5′H), 2 dihydroflavonol 4-reductases (DFR), 2 leucoanthocyanidin reductases (LAR), 1 anthocyanidin synthase (ANS) and 2 anthocyanidin reductases (ANR). Thus, for every step from PAL to ANR in the PA biosynthesis pathway (Figure 1), there was at least one encoding gene significantly up-regulated in brown fiber (Table 5).

**Table 5:** PA synthase genes identified in cotton fiber and their expressions in BCF and WCF.

PA synthase^1^	Unigene/EST	Gene in D genome	WCF(TPM)	BCF(TPM)	FDR	ExpressionChanges^2^
PAL	Ghi.2173	Gorai.002G248000	104.7	1564.32	1.31E-11	U
	Ghi.3957	Gorai.009G416300	1.37	15.38	2.77E-04	U
	Ghi.24914	Gorai.011G238400	2.74	14.69	4.30E-05	U
	ES851993	Gorai.007G373600	0.01	1.4	0.03	N
C4H	Ghi.4426	Gorai.013G271700	99.55	460.48	6.82E-12	U
	Ghi.9347	Gorai.011G207000	2.06	171.68	1.12E-04	U
4CL	Ghi.9697	Gorai.003G052100	12.68	261.53	4.64E-07	U
	Ghi.1797	Gorai.011G053700	6.52	2.8	0.09	N
	Ghi.21520	Gorai.009G005900	1.55	0.71	0.54	N
	Ghi.24129	Gorai.013G269100	0.01	0.7	0.34	N
CHS	Ghi.1443	Gorai.011G161200	22.31	1726.6	4.65E-12	U
	Ghi.10134	Gorai.005G035100	74.15	690.55	6.24E-12	U
	Ghi.24663	Gorai.011G161200	1.37	172.03	2.78E-04	U
	Ghi.2516	Gorai.009G339300	3.09	24.13	2.32E-05	U
	Ghi.6103	Gorai.006G000200	3.43	18.18	1.43E-05	U
	BQ410047	Gorai.009G339300	1.71	16.78	2.46E-04	U
CHI	Ghi.10760	Gorai.013G023400	30.18	122.73	0	U
	Ghi.7860	Gorai.012G014600	31.91	36.36	0.47	N
F3H	Ghi.17176	Gorai.008G062900	189.14	3230.73	5.54E-12	U
	Ghi.7987	Gorai.007G194700	0.01	4.55	5.71E-04	U
	CO123602	Gorai.008G062900	0.34	1.75	0.10	N
F3′5′H	Ghi.14535	Gorai.001G134900	96.11	781.81	0	U
	BM360843	Gorai.001G134900	3.26	158.39	5.80E-05	U
DFR	Ghi.9795	Gorai.009G200600	1.55	31.12	3.17E-04	U
	Ghi.9683	Gorai.010G008900	0.01	23.08	0	U
	BQ401793	Gorai.009G200600	0.01	1.05	0.07	N
LAR	Ghi.18163	Gorai.008G186500	38.45	597.2	0	U
	Ghi.17301	Gorai.008G285400	0.01	15.73	4.77E-13	U
	Ghi.17277	Gorai.008G186500	0.01	2.8	0.01	N
	Ghi.2177	Gorai.008G285400	0.01	0.7	0.37	N
ANS	Ghi.1234	Gorai.004G205900	0.01	7.34	3.56E-06	U
	DW488687	Gorai.011G289300	0.69	0.01	0.44	N
ANR	Ghi.8422	Gorai.009G175500	101.63	4898.19	1.15E-11	U
	Ghi.964	Gorai.009G175500	1.37	33.57	2.79E-04	U

1 PA synthases are abbreviated as in Table 1.

2 U, significantly up-regulated in BCF; N, not significantly changed.

**Figure 3:**
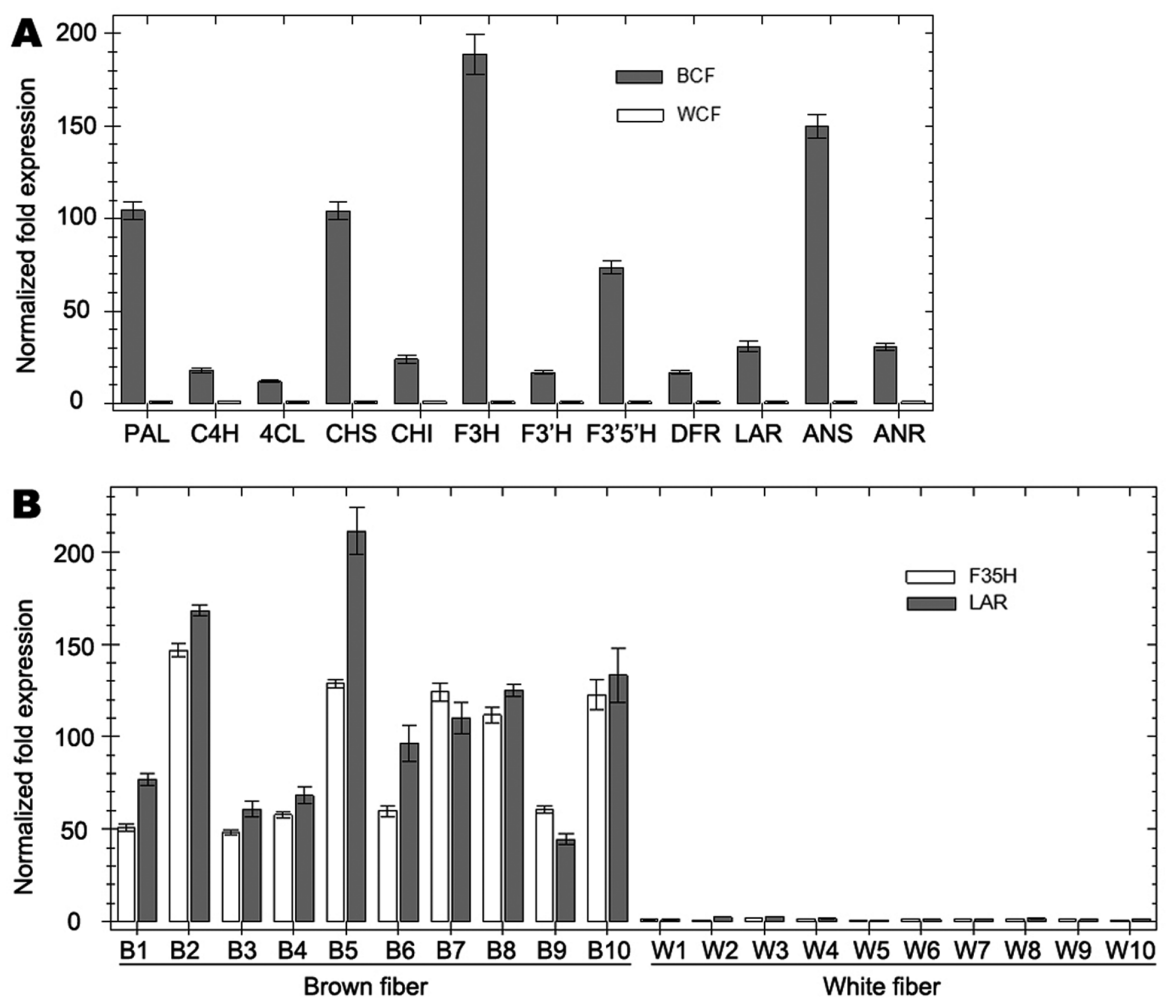
Detection of PA synthase genes in RILs by qRT-PCR. A, Comparison of expression levels of PA structural genes in white and brown fiber bulks (WCF and BCF, respectively). B, expression levels of LAR and F3′5′H genes in fibers of each 10 white and brown fiber RILs. Relative expression levels are normalized to cotton histone 3 and elongation initiation factor 5 genes. WCF and W1 are set as control in A and B, respectively.

To verify the result of DGE analysis, expression levels of the predominant PA synthase genes identified for all steps from phenylalanine to flavan-3-ols (Figure 1, Table 1 and 5) and a flavonoid 3′-hydroxylase homologous gene (F3′H, Gorai.008G198200 from *G. raimondii*) were detected in brown and white fibers via qRT-PCR. Compared to white fiber, all of the 12 PA synthase genes were up-regulated in brown fiber (16∼260 fold, Figure 3A). Furthermore, the high level expressions of PA synthase genes were co-segregated with brown fiber in individual RILs, as exemplified by LAR and F3′5′H genes (Figure 3B). These results were consistent with DGE profiles and confirmed that the brown fiber gene (*Lc1*) wholly activated PA biosynthesis pathway in cotton fiber.

**Figure 4:**
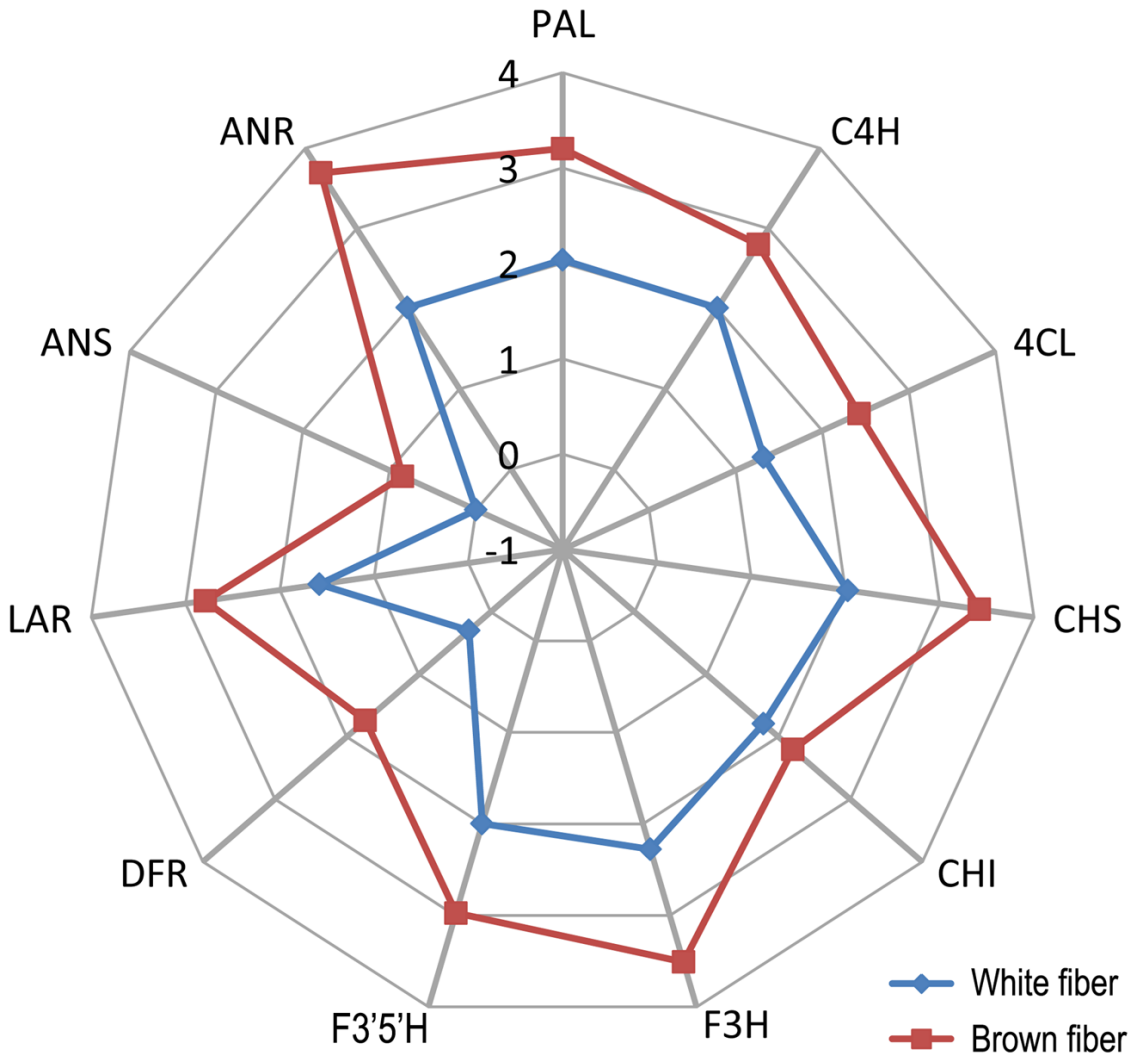
The transcription patterns of different PA synthases in brown and white fibers. Each point on the polygons represents a PA synthase and the scale corresponds to the transcript levels (log_10_TPM). PA synthases are abbreviated as in Table 1.

DGE analysis also revealed that transcript levels of different flavonoid synthases varied dramatically in cotton fibers (Table 5 and Figure 4). In white fiber, the synthases related to common steps in flavonoid biosynthesis (PAL, C4H, 4CL, CHS, CHI, F3H and F3′5′H) and PA specific steps (LAR and ANR) showed moderate expression levels (20∼200 TPM), while DFR and ANS had very low expression (<2 TPM). In brown fiber, the relative transcription pattern of different PA synthases was similar to that in white fiber (Figure 4). Although the whole PA pathway was significantly up-regulated in brown fiber, DFR and ANS had moderate (55.26 TPM) and low (7.34 TPM) expression levels, respectively, in comparison to much higher expression levels of other PA synthases (e.g. 940.2 TPM for F3′5′H and 616.43 TPM for LAR). These data suggested that the relative expression profile of different flavonoid synthase genes in cotton fiber was strictly regulated at transcription level.

### Monomeric Composition of PAs in Brown Cotton Fiber

**Figure 5:**
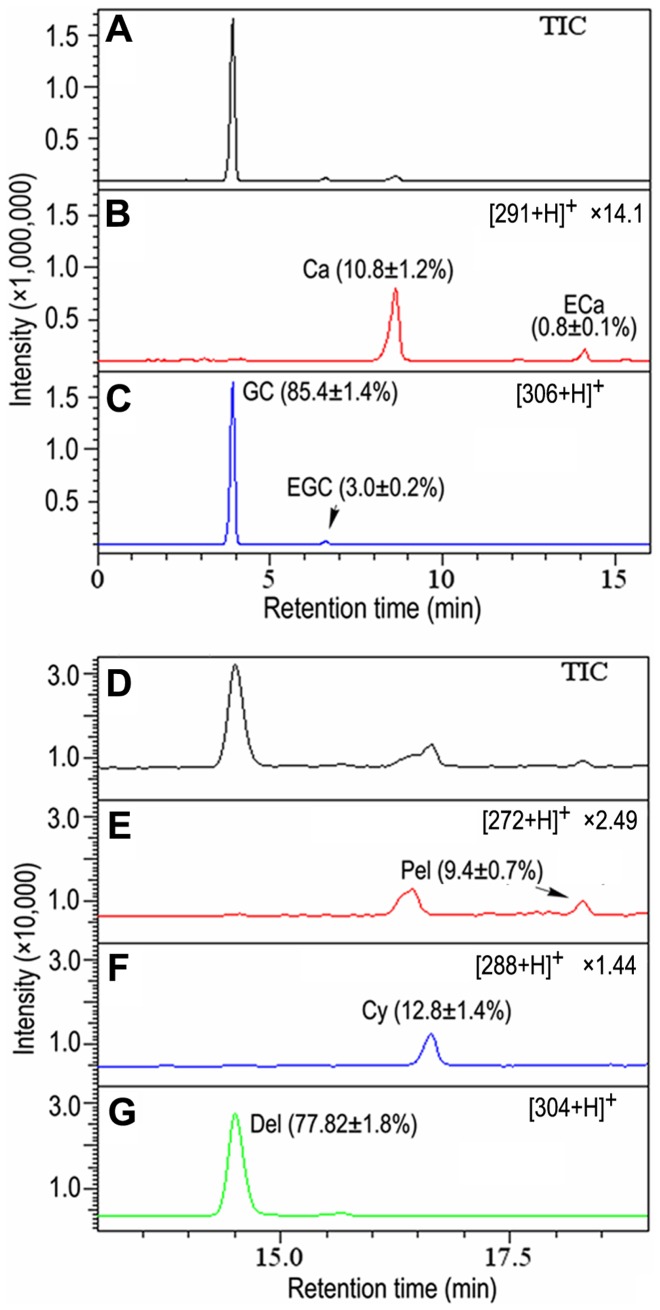
LC-MS analyses of PAs in brown cotton fiber. A, B and C, LC-MS profiles of monomeric flavan-3-ols in purified PAs showing total intensity count (TIC) and the intensity counts of [290+H]^+^ and [306+H]^+^, respectively. D, E, F and G, LC-MS profiles of anthocyanins in the PA acid hydrolysate showing TIC and the intensity counts of [272+H]^+^, [288+H]^+^ and [304+H]^+^, respectively. The mol percentages of flavan-3-ols or anthocyanidins are shown in average±SD for 3 duplicate tests. The profiles in B, E and F are magnified by folds as indicated. Ca, catechin; Cy, cyanidin; Del, delphinidin; ECa, epicatechin; EGC, epigallocatechin; GC, gallocatechin and Pel, pelargonidin.

Transcriptional analysis revealed a PA biosynthesis pathway wholly activated in brown cotton fiber. To further clarify the details in this pathway, we employed LC-MS method to determine the monomeric composition of PAs in brown fiber. Four flavan-3-ols (gallocatechin, epigallocatechin, catechin and epicatechin) were identified in the PAs from brown fiber by LC-MS analysis, with mol percentages of 85.4±1.4%, 3.0±0.2%, 10.8±1.2% and 0.8±0.1%, respectively (Figure 5A–C). Among these monomers, the 2, 3-trans-flavan-3-ols (gallocatechin and catechin) account for 96.2%, suggesting that most of free flavan-3-ols and then initiating units of polymeric PAs in brown cotton fiber are synthesized via LAR branch, rather than ANS/ANR (Figure 1).

The anthocyanidin composition in the acid hydrolysate reflected the extension unit composition of corresponding PAs [26]. To determine composition of PA extension units in brown fiber, we detected the anthocyanidins released by acid-butanol reactions. As shown in Figure 5D–G, three kinds of anthocyanidins (pelargonidin, cyanidin and delphinidin) were detected with the mol percentage of 9.4±0.7%, 12.8±1.4% and 77.8±1.8%, respectively. High percentage of delphinidin in PA acid hydrolysate indicated that the main PA extension units in brown cotton fiber were favan-3-ols trihydroxy1ated on B ring. Therefore, both initiating and extension units of PAs in brown cotton fiber consist mainly of flavan-3-ols trihydroxy1ated on B ring, indicating that F3′5′H plays a primary role in PA biosynthesis in brown cotton fiber.

Taken together, transcriptome and biochemical analyses collectively demonstrated a detailed PA biosynthesis pathway wholly up-regulated in brown cotton fiber, in which F3′5′H and LAR represented the primary flow for PA biosynthesis (Figure 1).

## Discussion

The exact chemical property of pigments is an important clue for exploitation of naturally colored cottons. Early extraction experiment suggested that pigments in naturally colored cotton belonged to flavonoids [1]. Hua *et al* revealed much higher PAL activity in brown cotton fiber compared to white fiber [9]. Expression analyses showed that several flavonoid synthase genes, such as CHI, F3H, DFR, ANS, ANR, C4H, CHS, F3′H and F3′5′H, were significantly up-regulated in brown fiber [6], [8]. Recently, Li and coworkers identified 15 flavonoid-related proteins (including PAL, CHS, F3H, DFR and ANR) with high abundance in brown cotton fiber via comparative proteomic analysis of BCF and WCF near isogenic lines [5]. In addition, the concentrations of PAs and PA precursors in brown fiber were much higher than in white fiber [5], [6], [8], [27]. These studies consistently indicated that the pigments in brown fiber belonged to PAs. In the present study, we aimed to dissect the details of PA biosynthesis pathway in brown cotton fiber. By DGE and qRT-PCR analyses, we found that all the investigated PA synthases (including PAL, C4H, 4CL, CHS, CHI, F3H, F3′H, F3′5′H, DFR, ANS, ANR and LAR) were significantly up-regulated in brown fiber, suggesting that the brown fiber gene (*Lc1*) activated the whole PA biosynthesis pathway in cotton fiber. Furthermore, biochemical analyses demonstrated that the main PA units were trihydroxylated on B ring and most of free flavan-3-ols were in 2, 3-trans form. These results demonstrated that F3′5′H and LAR represented the major flow for PA biosynthesis in brown cotton fiber. By dissecting the details of PA biosynthesis pathway in brown cotton fiber, our results paved the way to manipulate the biosynthesis of pigment and other flavonoids in cotton fiber via biotechnology techniques.

PAs are the predominant coloring compounds in seed coats, and may function as barrier to fungus infection of embryos [15]. It has been found for a long time that the PAs in cotton seed coats and fuzzes consist mainly of catechin and catechin-derived polymers [31]. Since the majority of PAs in brown cotton fiber are gallocatechin and its polymers, brown cotton fiber may have a different PA biosynthesis pathway independent of seed coat and fuzz. Additionally, with flavan-3-ols trihydroxylated on B ring as main units, brown cotton fiber may represent a novel PA resource compared to Arabidopsis, grapevine and *Medicago truncatula* which consist mainly of epicatechin and/or catechin [15]. Given the simplicity of cotton production, PAs from brown cotton fiber are also potential to be applied in food and medicine industry [12], [13].

In higher plant, PAs include a large number of oligomers or polymers of flavan-3-ols. In addition to various degrees of polymerization, difference in monomeric composition is a key factor influencing the complexity of PA components. There are two major branching points in the PA pathway which lead to different PA monomers (Figure 1). Firstly, DFR converts dihydrokampferol to leucoparlegonidin finally leading to PA monomers with a single hydroxyl on B ring, while F3′5′H catalyzes the hydroxylation on C-3′ and/or C-5′ of B ring which results in PA monomers di- or trihydroylated on B ring [32]. Secondly, LAR directly converts leucoanthocyanidins to 2, 3-trans-flavan-3-ols (catechin and gallocatechin), while ANS catalyzes leucoanthocyanidins to form anthocyanindins and then 2, 3-cis-flavan-3-ols (epicatechin and epigallocatechin) with ANR activity (Figure 1). High percentages of flavan-3-ols trihydroxylated on B ring implied that dihydrokampferols in brown cotton fiber were primarily converted to dihydromyricetin instead of leucopelargonidin and therefore F3′5′H activity should be much higher than DFR. Likewise, LAR activity might be much higher than ANS in brown cotton fiber, for the free flavan-3-ols were mainly in 2, 3-trans form. Consistently, DGE analysis revealed that the expression levels of F3′5′H and LAR were dramatically higher than those of DFR and ANS, respectively. These results implied that the flavonoid profiles in cotton fiber were mainly regulated by the relative expression pattern of corresponding synthases at transcription level.

Flavonoids may play roles in many aspects of plant growth and development [33], [34]. Several studies have suggested that pigment accumulation in cotton fiber may affect the fiber quality and yield [9], [27], [28]. Biochemical analyses indicated that the contents of PA and flavonoid precursors in brown cotton fiber were much higher than in white fibers [27]. Tan and coworkers showed that down-regulation of F3H and accumulation of flavonoid narigenin retarded the fiber development and reduced the final fiber quality and yield [27]. Our DGE analysis also showed that PA accumulation in developing cotton fiber might significantly affect several cellular components, KEGG pathways and other fiber-related physiological processes. However, the molecular basis of the negative influence of accumulation of pigment and other flavonoids on fiber quality and yield was largely unclear. Documenting the expression profiles of flavonoid synthases in white and brown fibers may facilitate to design transgenic strategy to engineer flavonoid pathway and to dissect the relationship between flavonoid accumulation and fiber development.

### Conclusion

Transcriptome analysis revealed that a whole PA pathway from phenylalanine to flavan-3-ol was activated in cotton fiber by the brown fiber gene. LC-MS analyses demonstrated that most of free favan-3-ols in brown cotton fiber were in 2, 3-trans form, and the main PA units were favan-3-ols trihydroxylated on B ring. The PA monomeric composition was consistent with the expression profiles of PA synthase genes, and suggested that F3′5′H and LAR represented the major flow of the PA biosynthesis pathway in brown cotton fiber.
